# Influence of Body Composition on Lung Function and Respiratory Muscle Strength in Children With Obesity

**DOI:** 10.14740/jocmr2382w

**Published:** 2015-12-28

**Authors:** Dirceu Costa Junior, Fabiana S. Peixoto-Souza, Poliane N. Araujo, Marcela C. Barbalho-Moulin, Viviane C. Alves, Evelim L. F. D. Gomes, Dirceu Costa

**Affiliations:** a Hortolandia City Hall, Sao Paulo, Brazil; b Postgraduate Course in Rehabilitation Sciences, University Nove de Julho, Sao Paulo, Brazil; c Postgraduate Program Federal University of Sao Carlos, Sao Carlos, Brazil

**Keywords:** Childhood obesity, Lung function, Respiratory muscle strength, Body composition, Respiratory system

## Abstract

**Background:**

Obesity affects lung function and respiratory muscle strength. The aim of the present study was to assess lung function and respiratory muscle strength in children with obesity and determine the influence of body composition on these variables.

**Methods:**

A cross-sectional study was conducted involving 75 children (40 with obesity and 35 within the ideal weight range) aged 6 - 10 years. Body mass index, z score, waist circumference, body composition (tetrapolar bioimpedance), respiratory muscle strength and lung function (spirometry) were evaluated.

**Results:**

Children with obesity exhibited larger quantities of both lean and fat mass in comparison to those in the ideal weight range. No significant differences were found between groups regarding the respective reference values for respiratory muscle strength. Male children with obesity demonstrated significantly lower lung function values (forced expiratory volume in the first second % (FEV_1_%) and FEV_1_/forced vital capacity % (FVC%) : 93.76 ± 9.78 and 92.29 ± 3.8, respectively) in comparison to males in the ideal weight range (99.87 ± 9.72 and 96.31 ± 4.82, respectively). The regression models demonstrated that the spirometric variables were influenced by all body composition variables.

**Conclusion:**

Children with obesity demonstrated a reduction in lung volume and capacity. Thus, anthropometric and body composition characteristics may be predictive factors for altered lung function.

## Introduction

Childhood obesity is recognized as one of the most prevalent public health problems in the Western world and is considered a worldwide epidemic, with tripled rates in developing countries in the last 20 years [1]. According to the latest census performed in Brazil, 33.5% of children and adolescents are overweight and 14.3% are obese [2].

Altered lung function in individuals with obesity is due to the excessive deposition of fat in the thoracic-abdominal region, which alters the mobility of the diaphragm muscles [3], thoracic expansion as well as lung compliance and strength, leading to a rapid, low amplitude breathing pattern with an increase in respiratory work and a reduction in maximum ventilatory capacity [4]. In a systematic review of the literature, Tenorio et al (2012) [5] demonstrated evidence of reduced lung volumes, such as forced vital capacity (FVC), forced expiratory volume in the first second (FEV_1_) and the FEV_1_/FVC ratio in children and adolescents with obesity.

Considering the evidence that obesity compromises the health of children and the lack of studies on this topic, particularly regarding the influence of body composition (specific measures of the percentage of lean and fat mass) on lung function, the present study is justified based on the investigation of such aspects to address the most relevant paradigms regarding the influence of early obesity on the future emergence of pulmonary, cardiovascular and metabolic disorders, thereby exploring elements that can assist in both treatment and prevention.

The hypothesis was that obesity leads to alterations in lung volumes and capacities and the strength of respiratory muscles in children without lung disease, but no knowledge on the influence of variables related to obesity and lung function or the relationship with gender has previously been established. Thus, the aim of the present study was to evaluate lung function and respiratory muscle strength among children with obesity and determine the influence of body composition and anthropometric characteristics on these variables.

## Methods

### Study population

A cross-sectional study was conducted involving 75 children aged 6 - 10 years. This study received approval from the Human Research Ethics Committee of University Nove de Julho (Brazil) under process number 285.499/2013. All legal guardians of the children evaluated signed a statement of informed consent.

The children were screened and evaluated at the municipal elementary school in the city of Hortolandia, State of Sao Paulo, Brazil, between June and October 2013. The inclusion criterion was respiratory health analyzed using a respiratory disease questionnaire (ATS-DLD-78-C) that has been adapted and validated for use in Brazil by pediatric pulmonologists [6]. This questionnaire has nine items addressing respiratory symptoms and a score of 7 or more points excluded the child. The other exclusion criteria were a history of premature birth (< 37 weeks), low birth weight (< 2,500 g), heart disease, neuromuscular disorder, abnormalities in the thoracic and/or abdominal regions that altered respiratory dynamics and cognitive impairment that rendered the understanding of the evaluation tests impossible.

### Anthropometric evaluation

The children remained in the quiet standing position, barefoot and wearing light clothing. Body weight was determined using a digital scale (Filizola^®^, Brazil). Height was determined using a stadiometer attached to the wall (Wiso) with resolution in millimeters. The body mass index (BMI) was determined as weight by height squared (kg/m^2^). Waist circumference was determined with a tape measure at navel level during expiration [7]. The Anthro plus program was used for the determination of z scores using the standards established by the World Health Organization (WHO, 2007) [8]. BMI z scores were used to classify the children as obese or within the ideal weight range. Z scores between 2 and -2 were considered ideal.

### Evaluation of body composition

A bioimpedance device (BIODYNAMICS MODELO 450; Biodynamics Corporation, Seattle, WA, USA) was used for the evaluation of body composition. The test was performed with four electrodes: two on the dorsum of the hands and two on the dorsum of the feet. A frequency of 50 kHz in alternating current passed through the input electrodes as the voltage passed through the body, which was measured using the output electrodes from which body impedance was derived. The variables analyzed through bioimpedance were fat mass and lean mass in kilograms and percentage [9].

### Evaluation of respiratory muscle strength

Respiratory muscle strength was evaluated using maximum inspiratory and expiratory pressures (MIP and MEP, respectively) using an analog pressure gauge (Critical Med, USA, 2002) with an operational interval of 0 to ±300 cm H_2_O, which was previously calibrated and equipped with an adaptor containing an air escape valve. The children were first given a demonstration on the correct performance of the tests to ensure that they maintained their lips firmly around the mouthpiece and compressed their cheeks to avoid the escape of air [10], employing the Black and Hyatt (1969) method [11].

MIP was measured during maximum inspiration at the level of total lung capacity, with inhalation beginning with the reserve volume and MEP was measured during maximum exhalation at the level of reserve volume, which began at the level of total lung capacity. The position reached at the end of the maximum efforts was maintained for at least 1 s for the characterization of the plateau pressure [12]. For these measures, the children were instructed to remain seated with their feet supported on the floor and a nasal clip in place.

All children performed at least three maximum inspiration and expiration efforts, with a 1-min interval between tests [13]. Maneuvers with no perioral leakage sustained for at least 1 s and with similar values (less than 10% difference) were considered technically acceptable. If a higher value was obtained on the third maneuver, the test was repeated until a value of ≤ 10% difference was obtained. Thus, the number of maneuvers could be more than three, although none surpassed five times. The highest value was recorded for the data analysis [12, 14]. The findings were compared to those predicted by Gomes et al (2014) [15].

### Evaluation of lung function

Lung function was evaluated using a spirometer (Easy-One, Medizintechnik, NDD AG^®^) with daily calibration prior to each exam following the recommendations of the American Thoracic Society [16] and the recommendations for lung function tests [17]. Slow vital capacity (SVC), FVC and maximum voluntary ventilation (MVV) were determined. The values were expressed as percentage of predicted based on the values established by Polgar and Promadhat (1971) [18].

### Statistical analysis

The Shapiro-Wilk test was used to determine the distribution of the data. Parametric data were expressed as mean and standard deviation and non-parametric data were expressed as median and interquartile range. The Student’s *t*-test (parametric variables) and the Mann-Whitney test (non-parametric variables) were used to compare anthropometric characteristics, body composition, muscle strength values and lung function between groups. The unpaired Student’s *t*-test was used for the comparison of MIP and MEP to predicted values. Pearson’s and Spearman’s correlation coefficients were calculated. Multiple stepwise regression analysis was used to establish significant associations between the independent variables and both lung function and respiratory muscle strength. All statistical analyses were performed using the BioStat program, version 5.0, with the level of significance set to 5% (P < 0.05).

## Results

A total of 75 children were analyzed (35 in the ideal weight range and 40 categorized with obesity). Table 1 displays the general characteristics, anthropometric data and bioimpedance data of the sample.

**Table 1:** Anthropometric Characteristics and Body Composition of Children With Obesity and Those in Ideal Weight Range Stratified by Gender

	Obesity	Ideal weight range	P
Male	n = 24	n = 16	
Age	7.75 ± 1.53	7.81 ± 1.27	0.22
Body mass (kg)	41.96 ± 8.21*	29.93 ± 7.08	< 0.0001
Height (m)	134.72 ± 7.98	132.93 ± 10.06	0.53
BMI (kg/m^2^)	23.10 ± 3.54	16.68 ± 1.88	< 0.0001
BMI z score	2.74 ± 0.60*	1.00 ± 0.57	< 0.0001
WC (cm)	79.56 ± 7.58*	62.12 ± 6.83	< 0.0001
Lean mass, kg	30.36 ± 5.76*	25.27 ± 4.56	0.0026
Fat mass, kg	11.75 (9.6 - 14.2)*	4.2 (9.6 - 14.2)	< 0.0001
Lean mass, %	71.45 (67.9 - 75.55)*	83.45 (83.17 - 89.17)	< 0.0001
Fat mass, %	28.55 (24.45 - 32.1)*	14.55 (10.82 - 16.22)	< 0.0001
Female	n = 16	n = 19	
Age	8.12 ± 1.5	8.33 ± 1.28	0.83
Body mass (kg)	43.93 ± 10.31*	29.5 ± 4.92	< 0.0001
Height (m)	132 ± 9.84	133 ± 8.95	0.83
BMI (kg/m^2^)	23.8 ± 3.18	16.44 ± 1.29	< 0.0001
BMI z score	2.59 ± 0.49*	0.49 ± 0.43	< 0.0001
WC (cm)	79.75 ± 8.82*	62.83 ± 4.04	< 0.0001
Lean mass, kg	29.6 (25.3 - 34.3)*	23.9 (20.7 - 28.7)	0.01
Fat mass, kg	12.6 (10.6 - 14.7)*	4.4 (3.1 - 5.37)	< 0.0001
Lean mass, %	71.4 (67.9 - 75.5)	85.4 (83.1 - 89.1)*	< 0.0001
Fat mass, %	30.1 (28.62 - 34.8)*	14.85 (10.15 - 18.15)	< 0.0001

Data expressed as mean ± standard deviation or median and interquartile range. BMI: body mass index; WC: waist circumference. *P < 0.05, significant difference between children with obesity and those in ideal weight range.

The bioimpedance analysis revealed greater lean mass (kg) and fat mass (kg) among the children classified as obese in comparison to those classified in the ideal weight range. However, the children in the ideal weight range had a greater percentage of lean mass and lower percentage of fat mass in comparison to those classified as obese. Table 2 displays respiratory muscle strength.

**Table 2:** Maximum Respiratory Pressures (MIP and MEP) Among Children With Obesity and Those in Ideal Weight Range

	Boys				Girls			
	MIP obtained (cm H_2_O)	MIP predicted (cm H_2_O)	MEP obtained (cm H_2_O)	MEP predicted (cm H_2_O)	MIP obtained (cm H_2_O)	MIP predicted (cm H_2_O)	MEP obtained (cm H_2_O)	MEP predicted (cm H_2_O)
Obese (n = 40)	70.8 ± 27.67	76.74 ± 11.42	72.00 ± 24.32	75.49 ± 11.17	72.5 ± 28.63	67.05 ± 9.93	71.25 ± 30.74	66.66 ± 9.41
Ideal weight (n = 35)	80.62 ± 21.43	70.34 ± 12.54	81.87 ± 18.24	77.78 ± 8.89	72.94 ± 19.68	60.44 ± 6.78	70.00 ± 16.48	65.55 ± 7.73

MIP: maximum inspiratory pressure; MEP: maximum expiratory pressure.

No significant difference in respiratory muscle strength was found between the children with obesity and those in the ideal weight range in relation to the predicted values for each gender or the group studied. With regard to lung function, the males in the group of children with obesity had significantly lower percentages of predicted FEV_1_ (93.76 ± 9.78) in comparison to the boys in the ideal weight range (99.87 ± 9.72). No significant differences were found regarding the other spirometric variables. No differences were found for any spirometric variables between the girls in the different groups (Table 3).

**Table 3:** Spirometric Measures of Children With Obesity and Those in Ideal Weight Range

	Obese	Ideal weight	P-value
Male			
SVC (%P)	99.41 ± 11.05	108.31 ± 12.77*	0.03
IRV (L)	0.93 ± 0.33	1.00 ± 0.44	0.10
IC (L)	1.51 ± 0.25	1.58 ± 0.43	0.30
EVR (L)	0.45 ± 0.27	0.56 ± 0.21	0.24
FVC (% P)	101.82 ± 12.07	103.87 ± 11.21	0.61
FEV_1_ (% P)	93.76 ± 9.78	99.87 ± 9.72*	0.04
FEV_1_/FVC (%P)	92.29 ± 3.38	96.31 ± 4.82*	0.002
MVV (%P)	73.23 ± 12.76	77.25 ± 18.08	0.46
Female			
SVC (%P)	96.81 ± 10.50	93.5 ± 8.18	0.31
IRV (L)	0.89 ± 2.66	0.83 ± 2.27	0.55
IC (L)	1.43 ± 0.34	1.30 ± 0.17	0.05
EVR (L)	0.47 ± 0.41	0.47 ± 0.24	0.96
FVC (%P)	96.81 ± 11.89	11.89 ± 8.98	0.86
FEV_1_ (%P)	89.87 ± 9.81	89.94 ± 9.13	0.98
FEV_1_/FVC (%P)	91.37 ± 6.94	93.5 ± 5.81	0.46
MVV (%P)	67.06 ± 14.01	73.5 ± 14.17	0.19

Data expressed as mean and standard deviation. SVC: slow vital capacity; IRV: inspiratory reserve volume; IC: inspiratory capacity; ERV: expiratory reserve volume; FVC: forced vital capacity; FEV_1_: forced expiratory volume in the first second; MVV: maximum voluntary ventilation. *P < 0.05, significant difference between obese and ideal weight groups.

Table 4 shows the regression models of the variables with the strongest associative contributions. The subscales of the spirometric variables were influenced by body mass, BMI, BMI z score, lean mass and fat mass and vice versa. Waist circumference also influenced FEV_1_ (R^2^ = 22.4; P = 0.003).

**Table 4:** Multiple Linear Regression Among Anthropometric Data, Body Composition, Spirometric Variables and Respiratory Muscle Strength

	SVC%		ERV		IRV		IC		FVC%		FEV_1_%		MVV%	
	R^2^	P	R^2^	P	R^2^	P	R^2^	P	R^2^	P	R^2^	P	R^2^	P
Body mass	20.36	0.001	17.63	0.01	24.13	0.001	15.38	0.006	32.20*	0.000	31.33	0.002	20.53	0.006
BMI (kg/m^2^)	20.25	0.000	17.48	0.008	22.54	0.001	15.39	0.001	32.17	0.000	28.65	0.000	17.30	0.001
BMI z score	10.38	0.005	10.58	0.004	11.96	0.002	23.49	0.007	18.92	0.000	19.14	0.000	12.83	0.002
Fat mass, kg	46.52*	0.000	24.96	0.005	0.38	0.001	15.30	0.000	56.67*	0.000	56.27	0.000	44.13	0.000
Fat mass, %	20.48	0.003	16.29	0.002	7.61	0.000	25.85	0.007	30.19	0.000	29.07	0.000	17.50	0.003
Lean mass, kg	30.03	0.000	20.22	0.014	47.04*	0.000	28.51	0.006	56.25*	0.000	34.36	0.000	21.81	0.008
Lean mass, %	23.06	0.002	16.33	0.005	19.62	0.000	18.81	0.013	30.49	0.000	31.24	0.000	17.56	0.003

SVC: slow vital capacity; ERV: expiratory reserve volume; IRV: inspiratory reserve volume; IC: inspiratory capacity; FVC: forced vital capacity; FEV_1_: forced expiratory volume in the first second; MVV: maximum voluntary ventilation.

## Discussion

Lung function was significantly influenced by body composition and vice versa, especially FVC and FEV_1_. Although Enright et al (1994) [19] had noticed that body composition, specifically lean mass, exerted a positive influence on respiratory muscle strength, the findings of the multiple linear regression analysis in the present study revealed that anthropometric and body composition were not predictors of respiratory muscle strength.

Lung function abnormalities are well documented in adults with obesity, who exhibit a reduction in volume and expiratory flow rate [20, 21]. In contrast, the few studies involving the pediatric population offer conflicting findings [22], which is what motivated the present investigation.

Spathopoulos et al (2009) [23] reported that the increase in BMI in children should be considered an important determinant in the reduction of spirometric variables. Although the FVC and FEV_1_ were significantly lower in male children with obesity, all children with obesity selected in the present study had spirometric variables within the range of normality, demonstrating no obstruction or respiratory restriction that could characterize a possible adverse lung and/or airway condition.

Studies have shown that weight gain is associated with reductions in FEV_1_ and VC, predisposing individuals with obesity to long-term adverse effects comparable to smoking and respiratory infection as well as occupational and environmental exposures [24]. Thus, it has become increasingly important to follow up lung function in children with obesity.

Ulger et al (2006) [25] evaluated 38 children with obesity and 30 children in the ideal weight range and found lower FVC and FEV_1_ in the former group. However, the authors reported the lack of reference values for the population studied (Turkish children) as a limitation of the respiratory function test.

No gender distinctions are made in a large part of studies involving children with obesity [23, 25-27]. However, males have greater lung function and respiratory muscle strength than females. Thus, the present sample was stratified by gender to allow a better discrimination of the data.

In a study conducted in Australia, Lazarus et al (1997) [27] investigated the effects of obesity on ventilatory function in children and found a negative association between weight and an increase in both FVC and FEV_1_, independently of height, age or gender. The authors put forth the hypothesis that large proportions of body fact are associated with diminished ventilator function. However, an important limitation to the study was the lack of a direct method for the evaluation of body composition.

In the present study, the multiple linear regression analysis revealed that anthropometric variables and body composition exerted a 10-56% influence on spirometric variables (Table 3). Such findings are in agreement with data described by Boran et al (2007) [26], who found that anthropometric measures exerted no significant effect on FEV_1_%, FVC% or the FEV_1_/FVC% ratio in the regression analysis.

Davidson et al (2014) [28] found a reduction in expiratory reserve volume with the increase in the BMI z score, but found no significant differences in this variable between children with obesity and those in the ideal weight range. However, the linear regression revealed that fat mass (in kg) is the variable that best predicts changes in expiratory reserve volume. Independently of an adverse airway condition, obesity can affect lung function in adults. Jones and Nzekwu (2006) [29] found that even a moderate increase in BMI was associated with a reduction in expiratory reserve volume in healthy adults.

The present findings on waist circumference are in agreement with data described by Chen et al (2009) [30], who found that this variable exerted an influence on the reduction in FEV_1_.

The lack of the longitudinal follow-up of lung function to investigate the effects of BMI on respiratory variables is a limitation of the present study. A high BMI is associated with an increased risk of future illness and mortality rates. Further studies are needed to determine whether weight loss and/or an increase in cardiopulmonary fitness is capable of improving lung function in children with obesity [28].

### Conclusion

Based on the present findings, children with obesity have lower lung volume and capacity. Moreover, anthropometric characteristics and body composition may be prediction factors of altered lung function, especially FVC and FEV_1_, which were mainly influenced by the percentage of body fat and the latter of which was also influenced by waist circumference.
